# A novel prognostic biomarker SPC24 up-regulated in hepatocellular carcinoma

**DOI:** 10.18632/oncotarget.5510

**Published:** 2015-10-21

**Authors:** Pengpeng Zhu, Junfei Jin, Yan Liao, Jun Li, Xue-Zhong Yu, Weijia Liao, Songqing He

**Affiliations:** ^1^ Laboratory of Hepatobiliary and Pancreatic Surgery, Affiliated Hospital of Guilin Medical University, Guilin, Guangxi, People's Republic of China; ^2^ Guangxi Key Laboratory of Molecular Medicine in Liver Injury and Repair, Guilin Medical University, Guilin, Guangxi, People's Republic of China; ^3^ Disease Prevention and Control Center of Guilin, Guilin, Guangxi, People's Republic of China; ^4^ Microbiology & Immunology, Medical University of South Carolina, Charleston, SC, USA

**Keywords:** hepatocellular carcinoma, SPC24, prognosis, proliferation, invasion

## Abstract

SPC24 is an important component of the nuclear division cycle 80 (Ndc80) kinetochore complex, which plays an essential role in the coupling of kinetochore to spindle microtubules (MTs) and the accurate segregation of chromosomes during mitosis. However, the functional role of SPC24 in hepatocellular carcinoma (HCC) remains unknown. Here, we detected the expression of SPC24 in HCC and analyzed its association with clinicopathologic features and prognosis of HCC patients. The expression of SPC24 mRNA was investigated in 212 cases of paired HCC and adjacent liver tissues by quantitative real-time PCR (qRT-PCR) and in the tissues of 20 HCC patients by semi-quantitative RT-PCR. Additionally, the expression of SPC24 protein was detected in 69 cases of HCC by immunohistochemistry (IHC) or in 2 cases of HCC tissues by Western-blotting. Furthermore, small interfering RNA (siRNA)-mediated silencing of SPC24 was employed in SMMC7721 and HepG2 human HCC cells to investigate cell proliferation, invasion and apoptosis. Survival curves were plotted using the Kaplan-Meier method, and differences in survival probability were obtained using the log-rank test. Independent predictors associated with disease-free survival (DFS) and overall survival (OS) were analyzed using the Cox proportional-hazards regression model. In this study, we showed that SPC24 was noticeably increased in HCC tissues compared to normal adjacent noncancerous tissues, at both mRNA and protein levels. High expression of SPC24 was significantly correlated with alpha-fetoprotein (AFP) (*p* = 0.044), median size (*p* = 0.030), tumor number (*p* = 0.019), and Barcelona-Clinic Liver Cancer (BCLC) stage (*p* = 0.015). Kaplan-Meier analysis showed that the DFS and OS of high SPC24 expression group was significantly shorter than that of low SPC24 expression group (*p* < 0.001; *p* = 0.001; respectively). The prognostic impact of SPC24 was further confirmed by stratified survival analysis. Importantly, multivariate analysis identified SPC24 upregualtion (*p* = 0.001), PVTT (*p* = 0.007), size of tumor > 5 cm (*p* < 0.001) as independent risk factors of DFS after resection, and SPC24 upregualtion (*p* < 0.001), PVTT (*p* = 0.029), size of tumor > 5 cm (*p* = 0.002), recurrence (*p* < 0.001) as independent prognostic factors for the OS of HCC patients. Additionally, siRNA-mediated silencing of SPC24 dramatically suppressed cell growth, adhesion, invasion and increased apoptosis in HCC cells. In conclusion, these results showed for the first time that SPC24 expression was significantly up-regulated in HCC, which may act as a novel prognostic biomarker for patients suffering from this deadly disease. Additionally, silence of SPC24 inhibiting HCC cell growth indicated that SPC24 may be a promising molecular target for HCC therapy.

## INTRODUCTION

Hepatocellular carcinoma (HCC) is one of the most malignant tumors with a steadied increasing incidence worldwide. The annually newly reported HCC cases were 740,000 and the death cases were 690,000, among which approximately 50% are in China [1, 2]. The major underlying causes for HCC cases are chronic viral hepatitis, alcohol abuse, cirrhosis, aflatoxin, genetic susceptibility and epigenetic changes [3–5]. Despite the improvements in treatment platforms including surgical resection, radiofrequency, transarterial therapy, chemotherapy and radiotherapy, the long-term survival remain dismal due partly to post-treatment relapse and distant metastasis [6, 7]. Therefore, it is of great importance for us to identify novel molecular markers that involved in the proliferation, invasion, differentiation and metastasis of HCC, and further improve the diagnosis and prognosis prediction of patients with HCC.

Genetic stability relies principally on the proper and accurate chromosome segregation during the development of eukaryotes; the heredity of too many or too few chromosomes can be dangerous and even deadly for the daughter cells, which may contribute directly to the evolution of cancer [8–10]. This high-fidelity of chromosome segregation is achieved through appropriate coordination among chromosomes, kinetochores, and spindles. The kinetochore, a large structure composed of multiple protein subcomplexes assembled on chromosomal domains of each sister chromatid pair, forms a dynamic interface between kinetochores and nuclear spindle microtubules (MTs) to enable faithful chromosome segregation during mitosis [11–13]. A functional kinetochore is consisted of inner kinetochore proteins that connect with centromeric DNA and outer kinetochore that interaction with spindle microtubules [14].

Nuclear division cycle 80 (Ndc80), one of the core components of outer kinetochore, is essential for the stable formation of kinetochore-microtubule anchoring and correct chromosome segregation during mitosis. The four-protein Ndc80 complex comprised of Ndc80 (also called Hec1 or KNTC2), Nuf2 (also called CDCA1), SPC24 and SPC25, which together form a dumbbell-like heterotramer [15]. The Ndc80 complex directly mediates microtubule binding by the Ndc80/Nuf2 heterodimer [16, 17], and the SPC24 and SPC25 heterodimer anchors the Ndc80 complex to the inner kinetochore [18, 19]. Malfunction in any of the four essential Ndc80 complex subunits may lead to high rates of chromosome missegregation and inactive forms of the spindle checkpoint in budding yeast, Caenorhabditis elegans, Schizosaccharomyces pombe, Xenopus laveis, and humans [20–23].

To the best of our knowledge, however, no population-based study has been made to examine the expression levels of SPC24 in pairs of human HCC tissues and HCC cell lines. Our study for the first time innovatively revealed that SPC24 is highly expressed in human HCC specimens as compared to adjacent noncancerous tissues. Furthermore, our results showed that the ability of proliferation and invasion was dramatically reduced via a small interfering RNA (siRNA)-mediated silencing of SPC24 in SMMC7721 and HepG2 human HCC cells. In line with these findings, an improved understanding the molecular mechanisms of SPC24 in tumor growth and metastasis will develop a novel candidate for molecule-targeted therapeutic strategies in HCC.

## RESULTS

### Elevated expression of SPC24 mRNA in human HCC

**Figure 1:**
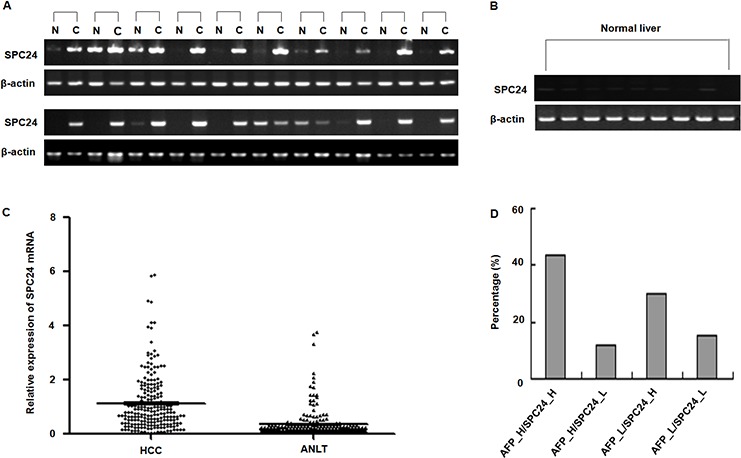
SPC24 mRNA expression in HCC specimens analyzed by RT-PCR and real-time RT-PCR **A.** The mRNA of SPC24 was measured in 20 cases of HCC specimens [C] and corresponding adjacent noncancerous liver tissues [N] by RT-PCR. The relative mRNA level of SPC24 was normalized based on that of an internal reference β-actin. PCR products were visualized by electrophoresis on 2% agarose gels. **B.** The mRNA of SPC24 was evaluated in 9 cases of normal liver tissues by semi-quantitative RT-PCR. β-Actin was used as an internal control. PCR products were visualized after electrophoresis through 2% agarose. **C.** The mRNA levels of SPC24 in 212 paired HCC specimens and adjacent noncancerous liver tissues (ANLT) were determined by quantitative real-time PCR, β-Actin was used as an internal control. **D.** Combined analysis of SPC24 mRNA expression and AFP level in examined 212 cases of HCC. The number represents the percentage among the 212 cases of HCC patients.

To compare the SPC24 mRNA in human HCC, we first tested the relative expression of SPC24 in HCC specimens using a semi-quantitative RT-PCR assay. As illustrated in Figure 1A, SPC24 mRNA levels were aberrantly elevated in 18 of the 20 analyzed HCC tissues (90%) in comparison to non-tumor controls, while the SPC24 mRNA was faintly expressed in 9 cases of normal liver tissues from hepatic hemangioma' surrounding liver tissues (Figure 1B). To further confirm these findings, the expression level of SPC24 was detected in 212 paired HCC tissue specimens by quantitative real-time RT-PCR. The data showed that SPC24 mRNA levels were increased in 73.1% HCC cases (155 in 212 cases), and decreased in 26.9% HCC cases (57 in 212 cases). The relative expression of SPC24 mRNA in HCC specimens was significantly higher than that in adjacent noncancerous livers (mean ± SD, 1.123 ± 0.072 *VS*. 0.350 ± 0.036, *and p* < 0.0001) (Figure 1C). These findings revealed that SPC24 was significantly up-regulated in HCC tissues.

### SPC24 and AFP were positively correlated in HCC

In order to improve diagnostic accuracy of HCC, we assessed whether combined determination of AFP and SPC24 was better than AFP alone. As shown in Figure 1D, the proportions of patients with high SPC24 expression along with raised serum AFP was 43.40% (92/212), patients who had positive AFP results but not up-regulation of SPC24 was 11.79% (25/212), and patients with up-regulation of SPC24 alone was 29.72% (63/212), suggesting combined measurement of AFP and SPC24 may increase HCC diagnostic accuracy.

### SPC24 protein was elevated in human HCC

**Figure 2:**
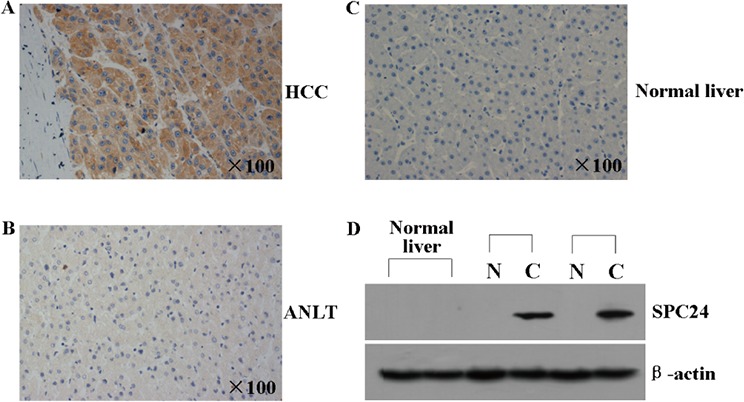
SPC24 protein was analyzed by immunohistochemistry and western blot in HCC tissues **A** and **B.** The representative immunohistochemical pictures of one pair of HCC specimen (upper panel) and corresponding noncancerous tissue (lower panel) from a tissue array containing 69 pairs of HCC specimens were shown. The pictures were stainied with SPC24 antibody, and the nuclei were counterstained with hematoxylin. **C.** The representative immunohistochemical staining in one normal liver tissue is shown. The nuclei were counterstained with hematoxylin. **D.** SPC24 expression in 2 normal liver tissues, 2 paired HCC [C] and adjacent non-cancerous liver tissues [N] was evaluated by Western blot. β-actin protein was used as an internal control.

To further evaluate the pattern of SPC24 protein expression, tissue microarrays containing 69 pairs of HCC were examined by immunohistochemical staining using a specific antibody against SPC24, the staining intensity was scored on a scale of 0–3. The data showed that none of the 9 normal liver tissues, 31.90% (22 out of 69) of adjacent noncancerous liver tissues (ANLT) showed positive for SPC24 staining, however, 73.90% (51 out of 69) of HCC tissues was stained positively by using SPC24 antibody (Figure 2A–2C). Consistent with above result, SPC24 protein expression analyzed by western blotting was highly expressed in HCC but faintly expressed in ANLT and normal liver tissues (Figure 2D).

### Correlations between SPC24 expression and clinicopathologic variables

To determine the clinical significance of SPC24 in HCC, the relationship between SPC24 expression and clinicopathological characteristics was analyzed. The data showed that SPC24 mRNA level was significantly higher in the group with AFP >200 ng/mL than that ≤200 ng/mL (χ^2^ = 4.046, *p* = 0.044), and it is higher in the group with diameter of tumor > 5 cm than that ≤5 cm (χ^2^ = 4.696, *p* = 0.030), and this tendency was also found in the group with multiple tumor nodules than that with a single tumor (χ^2^ = 5.461, *p* = 0.019). Furthermore, SPC24 expression was also associated with high BCLC stage (B-C) of HCC (χ^2^ = 5.919, *p* = 0.015). However, upregulation of SPC24 mRNA had no significant relationship with age, gender, family history, wine-drinking, HBsAg, cirrhosis, the presence of PVTT, distant metastasis, and recurrence (all *p* > 0.05, Table 1).

**Table 1:** Correlation between the clinicopathologic variables and SPC24 in HCC

Clinical character	Variable	No.of patients	SPC24		χ^2^	*p*-value
			low n (%)	high n (%)		
Age (years)	≤60	167	44 (26.3)	123 (73.7)	0.116	0.733
	>60	45	13 (28.9)	32 (71.1)		
Gender	Female	31	8 (25.8)	23 (74.2)	0.022	0.883
	Male	181	49 (27.1)	132 (72.9)		
Family history	No	177	44 (24.9)	133 (75.1)	2.243	0.134
	Yes	35	13 (37.1)	22 (62.9)		
Wine-drinking	No	100	24 (24.0)	76 (76.0)	0.802	0.370
	Yes	112	33 (29.5)	79 (70.5)		
HBsAg	Negative	37	8 (21.6)	29 (78.4)	0.632	0.427
	Positive	175	49 (28.0)	126 (72.0)		
AFP (μg/l)	≤200	95	32 (33.7)	63 (66.3)	4.046	**0.044**
	>200	117	25 (21.4)	92 (78.6)		
Median size (cm)	≤5	52	20 (38.5)	32 (61.5)	4.696	**0.030**
	>5	160	37 (23.1)	123 (76.9)		
Cirrhosis	No	20	7 (35.0)	13 (65.0)	0.739	0.390
	Yes	192	50 (26.0)	142 (74.0)		
Tumor number	Single	145	46 (31.7)	99 (68.3)	5.461	**0.019**
	Multiple	67	11 (16.4)	56 (83.6)		
BCLC stage	0-A	101	35 (34.7)	66 (65.3)	5.919	**0.015**
	B–C	111	22 (19.8)	89 (80.2)		
PVTT	No	166	46 (27.7)	120 (72.3)	0.264	0.607
	Yes	46	11 (23.9)	35 (76.1)		
Distant metastasis	No	193	51 (26.4)	142 (73.6)	0.234	0.629
	Yes	19	6 (31.6)	13 (68.4)		
Recurrence	No	145	41 (28.3)	104 (71.7)	0.450	0.502
	Yes	67	16 (23.9)	51 (76.1)		

Abbreviations: HBsAg, hepatitis B surface antigen; AFP, alpha-fetoprotein; BCLC, Barcelona clinic liver cancer; PVTT, portal vein tumor thrombus.

### Univariate analysis of the prognostic power of parameters in HCC patients

To further determine the potential prognostic significance of SPC24 in HCC patients, we performed univariate analysis for prognosis usingtraditional clinicopathologic variables. The results showed that high SPC24 expression (*p* < 0.001), size of tumor >5 cm (*p* < 0.001), multiple tumor number (*p* < 0.001), B-C of BCLC stage (*p* < 0.001), the presence of PVTT (*p* < 0.001), and distant metastasis (*p* = 0.034) were significantly associated with poor DFS rate in HCC patients. In addition, high SPC24 expression (*p* < 0.001), AFP > 200 ng/mL (*p* = 0.029), size of tumor >5 cm (*p* < 0.001), multiple tumor number (*p* < 0.001), B-C of BCLC stage (*p* < 0.001), the presence of PVTT (*p* < 0.001), distant metastasis (*p* = 0.043), and recurrence (*p* < 0.001) were negative prognostic factors for OS in patients with HCC after resection (Table 2).

**Table 2:** Association between SPC24, clinical parameters and disease-free survival/overall survival

Clinical character	Category	No.of patients	Disease-free survival (months)			Overall survival (months)		
			Mean	95% CI	*p* value	Mean	95% CI	*p*-value
SPC24	Low	57	56.41	47.50–65.32	**0.001**	58.54	49.98–67.09	**0.001**
	High	155	34.56	29.05–40.07		40.96	35.77–46.14	
Age (years)	≤60	167	40.26	34.81–45.71	0.665	45.04	39.89–50.18	0.580
	>60	45	42.99	32.37–53.61		47.99	38.22–57.76	
Gender	Female	31	43.06	31.99–54.14	0.243	54.92	43.26–66.57	0.125
	Male	181	39.74	34.56–44.92		44.17	39.26–49.08	
Family history	No	177	38.83	33.56–44.10	0.080	43.65	38.67–48.63	0.062
	Yes	35	50.71	39.09–62.33		56.18	45.62–66.75	
Wine-drinking	No	100	45.48	38.02–52.94	0.128	50.09	43.24–56.94	0.064
	Yes	112	37.22	30.95–43.48		41.89	35.90–47.88	
HBsAg	Negative	37	38.57	26.95–50.19	0.929	46.01	35.58–56.44	0.933
	Positive	175	41.12	35.77–46.48		45.67	40.61–50.73	
AFP (ng/ml)	≤200	95	45.90	38.59–53.20	0.053	51.73	45.09–58.37	**0.029**
	>200	117	36.67	30.27–43.07		40.82	34.72–46.92	
Tumor size (cm)	≤5	52	64.19	55.77–72.62	<**0.001**	67.49	60.09–74.89	<**0.001**
	>5	160	33.11	27.81–38.41		38.64	33.57–43.71	
Cirrhosis	No	20	30.79	15.58–45.99	0.159	36.15	22.12–50.18	0.221
	Yes	192	41.75	36.66–46.85		46.70	41.91–51.49	
Tumor number	Single	145	47.16	41.23–53.10	<**0.001**	51.84	46.39–57.29	<**0.001**
	Multiple	67	27.08	19.72–34.44		32.49	25.16–39.82	
BCLC stage	0-A	101	55.13	48.21–62.05	<**0.001**	60.06	54.00–66.12	<**0.001**
	B–C	111	27.96	22.16–33.76		32.66	26.93–38.39	
PVTT	No	166	46.43	40.85–52.02	<**0.001**	51.74	46.67–56.81	<**0.001**
	Yes	46	21.37	13.95–28.79		24.14	16.65–31.64	
Distant metastasis	No	193	42.26	37.14–47.39	**0.034**	47.05	42.26–51.83	**0.043**
	Yes	19	24.92	13.54–36.31		32.74	19.03–46.45	
Recurrence	No	145				35.11	29.82–40.41	<**0.001**
	Yes	67				68.58	62.90–74.25	

Abbreviations: HBsAg, hepatitis B surface antigen; AFP, alpha-fetoprotein; BCLC, Barcelona clinic liver cancer; PVTT, portal vein tumor thrombus; CI, confidence interval.

### Multivariate analysis of the prognostic power of parameters in HCC patients

Subsequently, to evaluate the independent impact of SPC24 expression in HCC prognosis, the above parameters showed significant statistically were subjected to multivariate Cox's proportional hazard regression analysis. High SPC24 expression (HR, 2.122; 95% CI, 1.335–3.374; *p* = 0.001), PVTT (HR, 1.872; 95% CI, 1.185–2.957; *p* = 0.007), size of tumor > 5 cm (HR, 2.892; 95% CI, 1.642–5.094; *p* < 0.001) were independent prognostic factors for DFS in HCC patients, and high SPC24 expression (HR, 2.510; 95% CI, 1.554–4.055; *p* < 0.001), PVTT (HR, 1.666; 95% CI, 1.053–2.636; *p* = 0.029), size of tumor >5 cm (HR, 2.431; 95% CI, 1.369–4.317; *p* = 0.002), and recurrence (HR, 2.694; 95% CI, 1.618–4.486; *p* < 0.001) were independent prognostic markers for OS (Table 3).

**Table 3:** Cox multivariate proportional hazard model of independent predictors on disease-free and overall survival

Variable	Disease-free survival		Overall survival	
	Hazard ratio (95% CI)	*p* value	Hazard ratio (95% CI)	*p*-value
SPC24, (high *vs* low)	2.122 (1.335–3.374)	**0.001**	2.510 (1.554–4.055)	<**0.001**
Tumor number (multiple *vs* single)	1.012 (0.638–1.607)	0.959	1.066 (0.673–1.687)	0.786
BCLC stage (B-C *vs* 0–A)	1.559 (0.889–2.732)	0.121	1.356 (0.773–2.379)	0.288
PVTT (yes *vs* no)	1.872 (1.185–2.957)	**0.007**	1.666 (1.053–2.636)	**0.029**
Tumor size, cm (>5 *vs* ≤ 5)	2.892 (1.642–5.094)	<**0.001**	2.431 (1.369–4.317)	**0.002**
Distant metastasis, (yes *vs* no)	1.353 (0.768–2.383)	0.296	1.739 (0.998–3.028)	0.051
Recurrence, (yes *vs* no)			2.694 (1.618–4.486)	<**0.001**
AFP, ng/ml (>200 *vs* ≤ 200)			1.083 (0.732–1.601)	0.691

Abbreviations: BCLC, Barcelona clinic liver cancer; PVTT, portal vein tumor thrombus; AFP, alpha-fetoprotein.

### Association between the survival status and SPC24 mRNA expression

**Figure 3:**
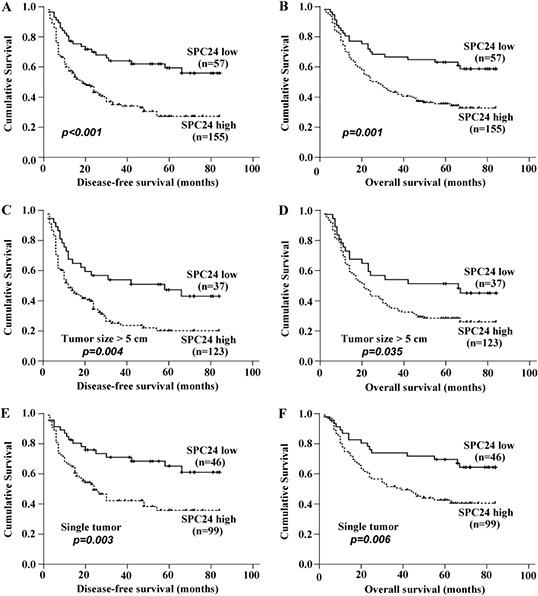
SPC expression affected disease-free survival rate and overall survival rate in patients with HCC All patients were divided into two groups according to the expression level of SPC24, then the prognostic significance of SPC24 was evaluated by Kaplan-Meier survival analysis and log-rank test. **A.** DFS of patients with high expression of SPC24 was shorter than that with low expression of SPC24. **B.** OS of patients with high expression of SPC24 was shorter than that with low expression of SPC24. **C** and **D.** High expression of SPC24 in HCC with diameter of tumor size > 5 cm appeared apparent prognostic value in predicting poorer DFS and OS. **E** and **F.** High expression of SPC24 in HCC patients with one single tumor nodule was significantly correlated with shorter DFS and OS.

Kaplan-Meier and log-rank survival tests showed that DFS and OS were significant different between high and low SPC24 mRNA expression groups among 212 HCC patients. The postoperative median DFS time in high SPC24 group was 34.56 months (95% CI, 29.05–40.07), significantly shorter than that of 56.41 months in low SPC24 group (95% CI, 47.50–65.32) (*p* < 0.001, Figure 3A). Furthermore, the postoperative median OS time in high SPC24 group was 40.96 months (95% CI, 35.77–46.14), which remarkably shorter than that of 58.54 months in low SPC24 group (95% CI, 49.98–67.09) (*p* = 0.001, Figure 3B).

### Prognostic values of SPC24 mRNA expression in different HCC subgroups

Stratified survival analysis was performed to further evaluate the prognostic value of SPC24 in specific subgroups of the patients. High SPC24 expression was found in 123 cases from the 160 (76.9%) of HCC patients with diameter of tumor >5 cm, that showed its apparent prognostic value in predicting poorer DFS (*p* = 0.004) and OS (*p* = 0.035) (Figure 3C, 3D). In addition, high SPC24 expression was found in 99 cases from the 145 (68.3%) of HCC patients with one single HCC tumor nodule, and significantly correlated with shorter DFS (*p* = 0.003) and OS (*p* = 0.006) (Figure 3E, 3F). Taken together, our data suggested that SPC24 is a sensitive clinical parameter predicting survival of HCC patients and can serve as a useful prognostic molecular marker for various HCC subgroups.

### Silence of endogenous SPC24 by siRNA suppressed HCC cell growth

**Figure 4:**
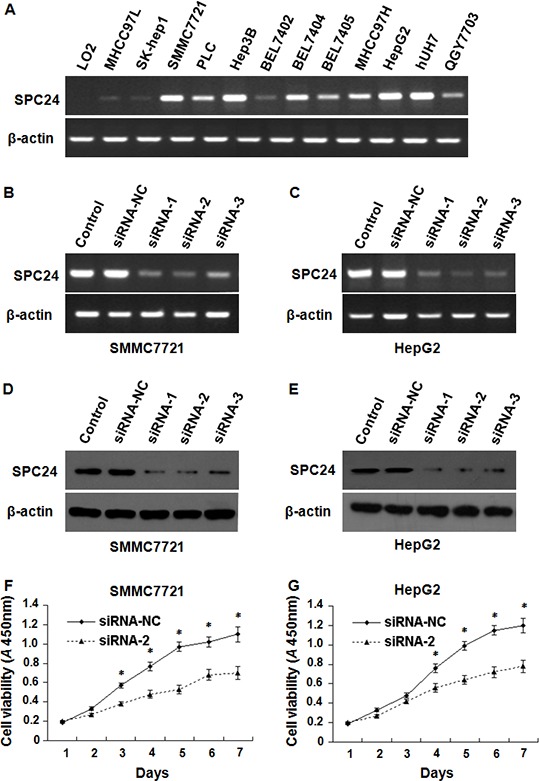
The inhibition of SPC24 decreased HCC cell growth SPC24 mRNA was detected by semi-quantitative RT-PCR; the products were visualized after electrophoresis on 2% agarose gels and β-actin served as an interval control. mRNA levels of SPC24 were detected by semi-quantitative RT-PCR in 12 kinds of human HCC cell lines and one of normal liver cell line **A.** RNAi efficiency was demonstrated by semi-quantitative RT-PCR in SMMC-7721 **B.** and HepG2 **C.** cells, and also confirmed by Western blot in SMMC-7721 **D.** and HepG2 **E.** cells. SPC24 inhibition by siRNA-2 dramatically decreased cell growth in both SMMC-7721 **F.** and HepG2 **G.** cells using cell viability assay. Experiments were performed in triplicate and data were shown as mean ± SD, *p < 0.05.

As above data showed that SPC24 expression is markedly higher in HCC tissues compared to non-tumor controls, we also tested SPC24 expression in human HCC cells by semi-quantitative RT-PCR. Figure 4A showed that the expression level of SPC24 was abundant in MHCC97L, SK-hep1, SMMC7721, PLC, Hep3B, BEL7402, BEL7404, BEL7405, MHCC97H, HepG2, Huh7, and QGY7703 HCC cells, but weak in LO2 normal liver cell line.

To investigate the effect of SPC24 on cell growth, three specific siRNAs against SPC24 (siRNA-1, siRNA-2, siRNA-3) were employed to inhibit endogenous SPC24, the siRNA-2 knocked down SPC24 most effectively among them demonstrated by semi-quantitative RT–PCR (Figure 4B, 4C) and Western Blotting (Figure 4D, 4E) in SMMC7721and HepG2 cells. Next we found that SMMC-7721 and HepG2 cell growth was inhibited by siRNA-2 (Figure 4F, 4G) indicating that SPC24 knockdown could significantly suppress cell growth.

### SPC24 promoted HCC cell adhesion and invasion *in vitro*

**Figure 5:**
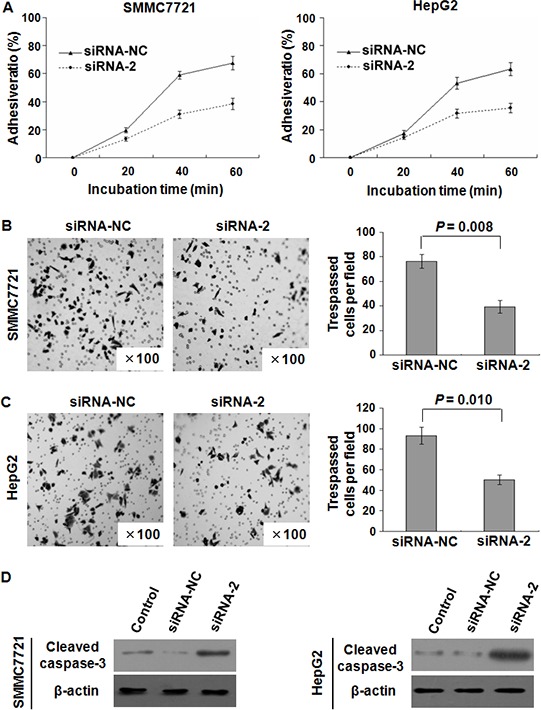
SPC24 inhibition affected HCC cell adhesion, invasion, and apoptosis SPC24 inhibition by siRNA-2 dramatically reduced cell adhesive ratio in both SMMC-7721 and HepG2 cells **A.** The invasiveness was evaluated by Matrigel assay in which scrambled siRNA served as a control and the cell number represented the mean values per field (from at least five fields) from 3 independent experiments (right panel) (mean ± SD), the data showed that the invasion of SMMC-7721 **B.** and HepG2 **C.** cells transfected with siRNA-2 was dramatically inhibited. Additionally, cleaved caspase-3 (active form of caspase-3) in SMMC7721 and HepG2 cells was increased by transfection with siRNA-2 confirmed by Western blot **D.**

The observation that endogenous SPC24 inhibition could significantly reduce cell growth prompted us to investigate HCC cell adhesion and invasion affected by SPC24. As shown in Figure 5A, the adhesive ability was inhibited in SMMC-7721 and HepG2 cells by siRNA-2. In addition, trans-well assay with Matrigel demonstrated that the invasiveness of the SMMC-7721 and HepG2 cells transfected with siRNA-2 was dramatically decreased compared to that with scrambled siRNA control ( *p* = 0.008, *p* = 0.010, respectively; Figure 5B, 5C). Taken together, these results indicated that SPC24 was a positive regulator of HCC invasion.

### Down-regulation of SPC24 induced cell apoptosis

As we know, caspase-3 was viewed as an executioner of apoptosis, cleaved caspase-3 protein in SMMC7721 and HepG2 cells transfected with siRNA-2 was determined, an increase of cleaved caspase-3 protein in above two cells transfected with siRNA-2 than that of scrambled siRNA control was observed (Figure 5D). This result suggested that down-regulation of SPC24 could induce cell apoptosis.

## DISCUSSION

The changes in chromosomal copy number (aneuploidy) and chromosome mis-segregation have been recognized as one of the most noticeable traits of cancers, which contribute directly to the development and metastasis of malignant tumor [8, 27]. Dysregulation of kinetochore may result in chromosome aneuploidy and instability, that is due to the defects in guiding proper and accurate chromosome segregation during mitosis [28–31]; cancer patients with chromosomal instability appeared significantly poor prognosis [32]. Therefore, better understanding of kinetochore-related genes can help us to know more about the pathogenic mechanism of cancer including HCC.

Kinetochore proteins were involved in cancers including HCC, for instance, the expression of Nuf2 was significantly enhanced in human HCC tissues compared with the corresponding adjacent normal tissues, lentivirus-mediated silencing of Nuf2 in HepG2 cells markedly suppressed short-term proliferation and long-term colony formation capacity, induced cell cycle arrest, and triggered cell apoptosis [33]. In Kaneko's report, mRNA of CDCA1, KNTC2, SPC24 and SPC25 was overexpressed in colorectal and gastric cancers compared with the corresponding normal mucosa, siRNA-mediated knockdown of either CDCA1 or KNTC2 significantly suppressed cell growth and induced HCC cell apoptosis [34].

As a core component of the Ndc80 kinetochore complex, SPC24 may play a crucial role in establishing and maintaining the kinetochore-microtubule attachment and metaphase alignment, it is essential for chromosomal directional movement to the spindle poles in anaphase [35]. The literature showed that a mutation of SPC24 may result in a chromosome segregation defect, due to premature spindle expansion and segregation of incompletely replicated DNA [36, 37]. However, the expression and biological function of SPC24 and its clinicopathologic/prognostic significance in HCC remain unclear.

This is the first report to validate and highlight the oncogenic role of SPC24 in HCC. We demonstrate that SPC24 mRNA and protein levels are significantly higher in human HCC tissues than corresponding adjacent noncancerous tissues, and this increase also exists in human HCC cell lines compared to normal immortal hepatocyte, indicating high SPC24 expression might be involved in the development of HCC. The correlation between high SPC24 expression and clinicopathologic variables was evaluated, higher SPC24 expression was positively associated with worse clinicopathologic characteristics including AFP >200 ng/mL, tumor size >5 cm, multiple tumor nodules, and high BCLC stage (B-C), all of which actually linked with the poorer prognosis in HCC, suggesting that SPC24 might be closely associated with the progression, invasion and metastasis of HCC. Of note, high SPC24 expression associated with adverse outcome and shorter survival implied that the change in SPC24 expression had a tremendous effect on kinetochore-microtubule attachment and alignment, which may lead to chromosome segregation defect, and further boost the progression and metastasis of HCC.

Tight connection between SPC24 expression and serum AFP level in HCC was further confirmed, the percentage of HCC patients with high SPC24 expression and raised serum AFP concentration was markedly higher than that in either alone. It is well known that AFP is the most commonly used marker for the diagnosis and monitoring of HCC, but its clinical value has been challenged due to the poor sensitivity and specificity [38]. Therefore, combined use of SPC24 and AFP elevated diagnostic accuracy for HCC patients, and might bring a significant impact on HCC clinical practice in future.

Our *in vitro* studies revealed down-regulation of SPC24 dramatically inhibited proliferation, adhesive ability and invasiveness of HCC cells, in which siRNA-mediated SPC24 inhibition might block stable attachment between kinetochore and microtubule that slow down tumor cell growth or stop cell dividing. Abnormal cell apoptosis play an important role in pathogenesis of many diseases including HCC [39–41], and caspase-3 is considered as a final executioner of apoptosis [42, 43]. This study revealed that down-regulation of SPC24 increased cleaved caspase-3 protein in SMMC7721 and HepG2 human HCC cells, demonstrating that the change of SPC24 expression might affect HCC cell apoptosis. As we know, siRNA-based drugs had proven to be a valuable strategy *in vivo* therapy [44, 45], this finding also provided a strong rationale to target SPC24 for effective HCC treatment.

Strong evidence confirmed that multiple tumors are an adverse prognostic factor in the patients with HCC [46]. Of note, the 5-year disease free outcome and overall survival rate in patients with single HCC tumor was significantly better than that of multiple tumor number [47, 48]. In addition, tumor size > 5 cm is an unfavorable prognostic factor in forecasting the prognosis of HCC patient [49, 50]. Furthermore, The appearance of PVTT seriously influenced liver vascular supply, contiguous with a wide dissemination of malignant tumor cells, thus resulting in intrahepatic metastasis and recurrences of HCC [51, 52]. Given that SPC24 was strikingly up-regulated in HCC tissues, it was closely associated with multiple tumor number, tumor size > 5 cm, and the presence of PVTT, we concluded that SPC24 might serve as a novel biomarker to predict prognosis outcome for HCC patients.

In summary, this study revealed SPC24 is overexpressed in human HCC tissues and cell lines, which might contribute to HCC progression and metastasis. More interestingly, our study validated high SPC24 expression as a new adverse independent prognostic factor in HCC. Therefore, the present study not only helps us to understand the role of SPC24 in the development and progression of HCC, but also strongly suggests that SPC24 might be a promising biomarker and a potential therapeutic target for the patients with HCC. Further study on larger patient population and *in vivo* validation of SPC24 in tumor growth and metastasis are highly warranted.

## MATERIALS AND METHODS

### Ethics statement

The survey protocol was performed according to the Helsinki Declaration and all procedures for informed consent, data collection and privacy protection were approved by the institutional ethics committee of Affiliated Hospital of Guilin Medical University. Written consent for using the samples for research purposes was obtained from either the patient or their family.

### Patient selection

A total of 212 patients who were diagnosed with HCC and underwent routine curative surgery between November 2001 and April 2007 at the Affiliated Hospital of Guilin Medical University (Guilin, Guangxi, China) were prospectively collected and retrospectively analyzed. All HCC patients were performed complete physical examination, hematologic and biochemistry profiles, ultrasonography (US), computed tomography (CT) scan, magnetic resonance imaging (MRI), and further defined on the basis of histopathology according to the American Association for the Study of Liver Diseases guidelines [24]. The tumor stage was confirmed according to the Barcelona Clinic Liver Cancer (BCLC) staging system [25]. The detailed clinicopathological variables of all patients, including age, gender, family history, wine-drinking, HBsAg, alpha-fetoprotein (AFP), median size, cirrhosis, tumor number, BCLC stage, presence of portal vein tumor thrombus (PVTT), distant metastasis, and recurrence were described in Table 1. In addition, nine specimens of normal liver tissues surrounding the hepatic hemangioma tissues were collected as normal liver tissues and verified by pathological examination. The HCC tissues samples were obtained immediately after surgical resection and were snap frozen in liquid nitrogen and placed at −80°C.

### Follow up

All of the 212 patients were regularly followed up by the trained clinical specialist through medical record review and on-site or phone interview. Patients received abdominal ultrasonography, chest radiography and serum AFP test every 6 months for the first two postoperative years and every 3–6 months thereafter. Patients were diagnosed with recurrence when ultrasonography, dynamic CT or MRI detected a new hepatic lesion. The mean postoperative follow-up period was 36.0 months (median, 21.0 months; range, 2.0 to 84.0 months). Disease-free survival (DFS) was measured from the date of surgery to the date of recurrence, metastasis, death or last follow-up. Overall survival (OS) was measured from the date of surgery to the date of death or last follow-up. We guarantee the clinical follow-up was not disclosed to laboratory personnel until statistical analysis.

### Cell lines and cell culture

One normal liver cell line LO2 and 12 liver tumor-derived cell lines were used in this study: MHCC97L, SK-hep1, SMMC7721, PLC, Hep3B, BEL7402, BEL7404, BEL7405, MHCC97H, HepG2, Huh7, and QGY7703, among them LO2, Huh7 and SMMC7721 were purchased from institute of chemistry and cell biology (Shanghai, China), and MHCC97L, SK-hep1, PLC, Hep3B, BEL7402, BEL7404, BEL7405, MHCC97H, HepG2 and QGY7703 were kindly provided by professor Hongsong Chen from Peking University People's Hospital, Peking University Hepatology Institute (Beijing, China). All cell lines were cultured in Dulbecco's modified Eagle's medium (DMEM; Gibco, Grand Island, NY, USA) containing 10% heat-inactivated fetal bovine serum (FBS) (Biowest, Nuaillé, France), 100 U/ml penicillin, and 100 mg/ml streptomycin (Hyclone). All the cell lines were maintained at 37°C under a humidified atmosphere consisting of 95% air and 5% CO_2_.

### RNA preparation and first-strand cDNA synthesis

Total RNA was extracted from HCC cell lines or frozen samples using Trizol regent (Invitrogen, Carlsbad, CA, USA) according to the protocols recommended by the manufacturer. The concentrations of extracted RNA were measured by the ratio of OD260/OD280 using an ultraviolet spectrophotometer, and the quality of which was judged by visualization of the 28S and 18S ribosomal RNAs on a 1.2% denaturing agarose. 2 μg total RNA was reverse-transcribed into cDNA with random primers using the Prime Script RT Reagent Kit (TaKaRa, Otsu, Japan) as per the manufacturer's instructions. Then the first-strand cDNA was stored at −20°C until use.

### Reverse transcription PCR (RT-PCR) analysis

For amplification of the matched mRNA of SPC24, the cDNA was performed using the primer pair designed specifically (forward: 5′-CCCAGAGCCTTC TCAATGCGA-3′; reverse: 5′-GGCTCACACTCATAAT CCCACT-3′). The length of the amplified fragment was 145 bp. The β-Actin mRNA level was used to standardize the measurements of the target gene using the specific primer pair (forward: 5′-GACAGGATGCAGAAGGAGATTACT-3′; reverse: 5′-TGATCCACATCTGCTGGAAGGT-3′), which generated a 142 bp fragment. PCR amplification was performed in 15 μL using a TaKaRa PCR Kit with the following amplification program: pre-denaturation for 5 min at 94°C, denaturation for 30 sec at 94°C, annealing for 30 sec at 55°C, extension for 30 sec at 72°C, and a final elongation step at 70°C for 5 min. PCR was performed for 28 or 32 cycles (β-actin 28 cycles; SPC24 32 cycles). After PCR, 5 μl of PCR products were resolved on a 2% agarose gel electrophoresis and visualized by ethidium bromide staining for 20 min.

### Quantitative real-time reverse transcription PCR (qRT-PCR) analysis

Quantitative real-time PCR (qRT-PCR) was performed following the manufacturer's instructions of SYBR Premix Ex Taq. Relative expression levels of SPC24 mRNA were compared to the levels of β-actin as the internal control. Gene-specific amplification was performed using an ABI Prism 7500 Sequence Detector System (Applied Biosystems, Foster City, CA, USA) with 15 μl SYBR Green PCR Master Mix (Applied Biosystems, Foster City, CA, USA) following the manufacturer's instructions. The optimized amplification protocol consisted of an initial denaturation step at 95°C for 10 min, followed by 40 cycles of denaturation at 95°C for 30 sec, annealing at 55°C for 30 sec and extension at 72°C for 30 sec, and fluorescence acquisition at 72°C. Relative SPC24 mRNA expression was calculated according to our previous report [26].

### Immunohistochemistry (IHC)

The sections from paraffin-embedded blocks were deparaffinized in xylene, then rehydrated in a graded ethanol series. Antigen retrieval was performed by microwaving for 3 minutes in citrate-buffered solution, pH = 6.0. Tissues were washed with phosphate buffered saline (PBS) for 3 times for 3 minutes, and the next endogenous peroxidase activity was quenched by incubation for 20 minutes in 3% hydrogen peroxide. For the purpose of preventing nonspecific staining, the sections were preincubated with 10% goat serum at room temperature for 30 minutes. Sections were incubated with an anti-SPC24 rabbit Ab (1:200 dilution, lot GR147853–1, Abcam) overnight in a humidified container at 4°C, the next day, after being washed with PBS, a secondary goat anti-rabbit antibody conjugated to horseradish peroxidase was applied for 1 hour at room temperature. Finally, the sections were stained with 3, 3-diaminobenzidine tetrahydrochloride (DAB) for detection and were then counterstained with hematoxylin. A negative control was obtained by replacing the primary antibody with normal rabbit serum. Semi-quantitative immunohistochemical detection was used to determine the SPC24 protein levels by two independent pathologists that were blinded to the patients' clinical and biochemical information, and the stained tissue sections were analyzed based on the 4-μm-thick sections as follows: the percentage of positive cell, grades 0–3 (0, no positive cells; 1, <25% positive cells; 2, 25%-50% positive cells; 3, >50% positive cells).

### Western blotting

Aliquots of purified proteins in the nucleus of HCC tissue were separated by electrophoresis on 12% SDS-polyacrylamide gels and electro-transferred onto immunoblot polyvinylidene difluoride (PVDF) membranes (Amersham, GE Healthcare, New Jersey, USA). The membrane was blocked with 5% skim milk in Tris buffered saline (TBS) with Tween (TBST; TBS plus 0.1% Tween 20) for 1 hour and then incubated overnight with rabbit polyclonal anti SPC24 antibody (1:1000 dilution, lot GR147853–1, Abcam) or cleaved caspase-3 rabbit antibody (1:500 dilution, lot 23, Cell Signaling) at 4°C. β-actin was used as internal positive control. The next day, after 3 washes in TBST for 15 min, the membranes were incubated with affinity purified HRP-conjugated goat anti-rabbit secondary antibody for 1 hour at 37°C. Blots were visualized by enhanced chemiluminescence, detected on X-ray films (Fuji films). Three independent experiments were done.

### RNA interference

Small interference RNAs (siRNAs) were designed on the Whitehead Institute Web Server (http://jura.wi.mit.edu/bioc/siRNAext/) and chemically synthesized (Shanghai GenePharma Co). Three siRNAs against SPC24 were designed and their sequences were as follows: siRNA-1 (5′-GAGCCUUCUCAAUGCGAAGTT-3′ and 5′-CUUCGCAUUGAGAAGGCUCTT-3′), siRNA-2 (5′-CC GAGAAGCAGCUGCGAGATT-3′ and 5′-UCUCGCAGC UGCUUCUCGGTT-3′), and siRNA-3 (5′-UACCACCAA GUUAGUAAAATT-3′, and 5′-UUUUACUAACUUGGU GGUATT-3′). The control scrambled siRNA used was the following sequences: (5′-GAGUUAAAGUCAAAGU GACTT-3′ and 5′-GUCACUUUGACUUUAACUCTT-3′). For the purpose of warranting the synthesized sequences would not target other gene transcripts, BLAST searches of the human genome database were performed.

### Cell transfection

SMMC7721 cells (5 × 10^5^/well) and HepG2 cells (5 × 10^5^/well) were seeded in 6-well plates and cultured in complete medium until they became 60–80% confluent, then transiently transfected with specific siRNAs or scrambled siRNA at a final concentration of 50 nM in serum free media by Lipofectamine™ 3000 (Invitrogen life technologies, CA, lot no: 1576643) according to the manufacturer's protocol. After 6–8 hours incubation at 37°C in 5% CO_2_, 20% complete medium was added to the cells. Analysis of genes expression was performed 48 hours and 72 hours post transfection.

### Cell proliferation assay

To evaluate the effect of siRNA-mediated silencing of SPC24 on HCC cell proliferation, Cell Counting Kit-8 (CCK-8; Dojindo Laboratories, Japan) assay was performed in both SMMC7721 and HepG2 cells *in vitro* following the manufacturer's instructions. SMMC7721 cells (2 × 10^3^/well) and HepG2 cells (2 × 10^3^/well) were seeded into 96-well plates respectively. After washing with PBS, then 10 μl of CCK-8 solutions was added to each well and incubated for 1 h. Cell proliferation was determined by measuring the absorbance at 450 nm. All experiments were performed in triplicate.

### Cell adhesion assay

The 96-well plates were coated with 50 μL fibronectin dilutions or medium and placed in an incubator at 4°C overnight. For the purpose of blocking, after washing with PBS, each well was added with 150 μl 1% BSA and placed in the incubator at 37°C for 1 h, and then washed with pre-warmed serum-free medium that used for cell inoculation. SMMC7721 cells (3 × 10^3^/well) and HepG2 cells (3 × 10^3^/well) exposed to specific or scrambled siRNA for 48 hours were suspended and added into the 96-well plates respectively. After the non-adhered SMMC7721 and HepG2 cells were washed with PBS, cell adhesion ability was measured using the CCK-8 and assessed by reading the absorbance at a wavelength of 450 nm. Experiments were repeated three times and data were summarized as mean ± SD. The adhesive ratio was calculated with the following formula: (The OD 450 values of the attached cells/The OD 450 values of the total cells) × 100%.

### Cell invasion assay

Tumor cell invasion was measured *in vitro* using a transwell insert (Falcon 354480; BD Biosciences). For the purpose of hydrating the matrigel layer, the upper chamber of a transwell cell culture insert were coated with 100 μL serum-free DMEM medium and placed in an incubator at 37°C for 1–2 hour. After transfected with specific or scrambled siRNA for 24 hours, SMMC7721 and HepG2 cells were harvested, and cells were suspended with serum-free DMEM medium to the concentration of 1 × 10^5^ cells/mL. Then, 100 μL of cells suspension was immediately added to the upper chamber and 600 μL DMEM medium with 20% FBS to the lower chamber. For the control, medium containing 1% FBS was added to the lower chamber. The cells were then incubated at 37°C for 20 hours. The cells that invaded through the membrane to the bottom chamber were fixed in 4% paraformaldehyde and stained with 0.5% crystal violet for 20 min. Six high power fields (magnification, × 100) were randomly photographed and counted in each chamber to observe the cells. Each experimental group was repeated three times, and the means and standard deviations of numerous values were calculated.

### Statistical analysis

The statistical analyses were carried out using the SPSS13.0 (SPSS Inc, Chicago, IL). The association of SPC24 expression and clinicopathological parameters were analyzed using the Pearson χ2 test. Survival was determined using the Kaplan–Meier method, and survival curves between different groups were calculated with the log-rank test. After the univariate analysis, only variables with *p*-value < 0.05 were used in the multivariate analysis using the Cox proportional hazards model to identify the independent prognostic factors for DFS and OS. *P*-values less than 0.05 were considered statistically significant.
